# PON-1 and PON-2 Polymorphisms and PON-1 Paraoxonase Activity in People Living with HIV-1

**DOI:** 10.3390/antiox14020209

**Published:** 2025-02-12

**Authors:** Cadiele Oliana Reichert, Débora Levy, Luciana Morganti Ferreira Maselli, Joel da Cunha, Sandra Fátima Menosi Gualandro, Sérgio Paulo Bydlowski

**Affiliations:** 1 Lipids, Oxidation, and Cell Biology Group, Laboratory of Immunology (LIM19), Heart Institute (InCor), Hospital das Clínicas HCFMUSP, Faculdade de Medicina, Universidade de Sao Paulo, Sao Paulo 05403-900, SP, Brazil or cadiele@usp.br (C.O.R.); d.levy@hc.fm.usp.br (D.L.); lu_morganti@yahoo.com.br (L.M.F.M.); joelbioq@gmail.com (J.d.C.); 2 Department of Hematology, Hemotherapy, and Cell Therapy, Faculdade de Medicina FMUSP, Universidade de Sao Paulo, Sao Paulo 05419-000, SP, Brazil; sandrafmg@uol.com.br; 3 Instituto Nacional de Ciencia e Tecnologia em Medicina Regenerativa (INCT-Regenera), CNPq, Rio de Janeiro 21941-902, RJ, Brazil

**Keywords:** ARV, HIV-1, lipids, paraoxonase, PON-1, PON-2

## Abstract

Antiretroviral therapy (ART) has significantly improved the life expectancy of people living with HIV-1 (PLWH). However, prolonged ART use is linked to metabolic alterations and oxidative stress. The paraoxonase (PON) enzymes, especially PON-1 and PON-2, are critical in maintaining antioxidant balance. Their activity can be influenced by polymorphisms such as Q192R and L55M in PON-1 and A148G and S311C in PON-2. This study examines the impact of these polymorphisms on paraoxonase activity, lipid metabolism, and infection markers in PLWH under various ART regimens. This is a case-control study with 525 participants, 175 healthy controls (HC) and 350 PLWH divided into subgroups: T0 (ART-naïve, *n* = 48), T1 (ART with reverse transcriptase inhibitors, *n* = 159), and T2 (ART with protease inhibitors, *n* = 143). Paraoxonase activity was higher in PLWH (123.0; IQR: 62.0–168.0) compared to HC (91.0; IQR: 48.0–136.0, *p* < 0.001) but similar between HC and T0 (*p* = 0.594). T1 (125.0; IQR: 65.5–166.0) and T2 (123.0; IQR: 61.0–182.0) showed higher activity than HC (*p* = 0.002 and 0.003). Among 61 complete genotypes, 13 were unique to PLWH and 6 to HC (*p* < 0.001). L55L was more frequent in HC (49.7% vs. 36.9% in PLWH), while M55M was higher in PLWH (*p* = 0.004). The S311C genotype was more frequent in HC (39.2%) than PLWH (24.9%) (*p* = 0.003). The L55L genotype conferred 59.9% protection against HIV-1 (OR: 0.401; 95% CI: 0.228–0.704), while the M allele increased susceptibility by ~69% (OR: 1.694; 95% CI: 1.173–2.446). The M55M genotype and/or M allele may be linked to HIV-1 susceptibility. Prolonged ART use elevates PON-1 activity in PLWH.

## 1. Introduction

The mechanisms associated with the oxidative balance in the human body act as a seesaw between oxidant and antioxidant factors [1]. This balance maintains physiological homeostasis through the action of antioxidant agents, which can neutralize and/or metabolize reactive species [2,3]. Some of the most effective mechanisms against the increase in both cellular and tissue oxidative stress (OS) are antioxidant enzymes, among which the paraoxonase (PON) family is of importance [4,5,6]. The PON enzymes are composed of three isoforms, paraoxonase-1 (PON-1, EC 3.1.1.2, 3.1.1.81, 3.1.8.1), paraoxonase-2 (PON-2, EC 3.1.1.2, 3.1.1.81), and paraoxonase-3 (PON-3, EC 3.1.1.2, 3.1.1.81, 3.1.8.1), showing 52.4% sequence identity and 76.3% sequence similarity among the isoforms. The enzymes are located adjacent to human chromosome 7 (7q21-23) [7,8].

Although the three paraoxonase enzymes share a homologous protein structure, they are known to differ in their localization, function, and activity [9]. PON-1 is a calcium-dependent glycoprotein 43 kDa with 355 amino acid residues, synthesized in the liver [9]. In humans, the PON-1 enzyme is mainly associated with high-density lipoprotein 3 (HDL3) and in lower concentrations in very-low density lipoprotein (VLDL) and chylomicrons [10]. A small fraction of PON-1 can be found free in plasma [10]. PON-1 has esterase activity (arylesterase and lactonase), through the hydrolysis of aromatic ester compounds and aromatic and aliphatic lactones, and paraoxonase activity, through the hydrolysis of toxic oxonium metabolites [11,12,13,14].

The paraoxonase activity of the PON-1 enzyme can be regulated by environmental factors, different pathophysiological conditions, both genetic and epigenetic alterations, and the presence of single nucleotide polymorphisms (SNPs) in the regulatory region of the gene [15,16]. PON-1 exists in two isoforms, resulting from amino acid substitutions: glutamine (Gln) is replaced by arginine (Arg) at position 192, Q192R, and leucine (Leu) is substituted by methionine (Met) at position 55, L55M. These genetic polymorphisms have been linked to variations in PON-1 activity, either reduced or elevated, and the risk of developing certain diseases [17,18,19,20].

The PON-2 enzyme has approximately 40–43 kDa and is composed of 355 amino acids [18]. PON-2 is ubiquitously expressed intracellularly, particularly in the perinuclear region, endoplasmic reticulum, and mitochondria. The role of the PON-2 enzyme in the defense system against oxidative stress, and therefore apoptosis, is due to its high lactonase activity [21,22,23]. PON-2 has two SNPs that have clinical relevance. The A148G polymorphism, in which an alanine residue is exchanged for glycine at position 148 of the protein, and the S311C polymorphism, in which a serine is exchanged for a cysteine at position 311 [24]. Some studies associate the presence of both polymorphisms with the development of diseases related to increased oxidative stress, changes in lipid metabolism, type 2 diabetes, infection, and chronic inflammation with an antioxidative stress activity, but there is disagreement between the studies [25,26,27,28].

Human immunodeficiency virus type 1 (HIV-1) infection has been a subject of study since its discovery nearly four decades ago. Over the years there have been effective biotechnological advances in the diagnosis and introduction of antiretroviral therapy (ART), and people living with HIV (PLWH) have delayed the natural progression of the disease, wherein no disease symptoms appear and there is a decrease in co-infections and mortality and an increase in lifespan [29,30,31].

The World Health Organization (WHO) recommends a combination of different ARTs such as nucleotide and non-nucleotide reserve transcriptase inhibitors associated with each other or with protease inhibitors. The ART objective is to combat intense viral replication, which leads to a decrease in CD4 T lymphocytes (CD4-TLs). Such multi-drug combinations bring about significant improvements in the immune systems of PLWH. On the other hand, chronic use of antiretroviral therapy induces a prolonged pro-inflammatory state that is closely related to the development of chronic degenerative diseases caused, in part, by metabolic alteration, increased oxidative stress, and genomic instability [32,33,34].

The prolonged use of ART in PLWH has been associated with an increased incidence of comorbidities in this population. These alterations may also be associated with oxidative stress and genetic alterations resulting from the toxic effects of ART drug use and/or metabolism [35,36]. The presence of reactive species can negatively impact lipid metabolism, including lipoprotein dysfunction and lipid peroxidation [37,38]. This can exacerbate the dyslipidemia already linked to prolonged ART use, leading to changes such as increased triglyceride levels, reduced HDL cholesterol (HDL-C), and elevated low-density lipoprotein cholesterol (LDL-C). These metabolic changes heighten the risk of early atherosclerosis and cardiovascular diseases and lipodystrophy [39,40,41]. Given these characteristics, many studies have focused on improving the metabolic and antioxidant status of PLWH [42,43,44]. However, few studies have examined PON function in PLWH. In a study involving 624 PLWH, PON-1 paraoxonase activity was lower in ART-naïve individuals compared to those on ART and showed a positive correlation with the absolute number of CD4+ TL, suggesting a relationship with the immune system in PLWH [45].

The diverse functions of PON-1, including its antioxidant and anti-inflammatory roles, as well as its involvement in lipid metabolism, make this enzyme particularly attractive for studies focused on metabolic disorders and conditions related to oxidative stress, mainly because it is considered a cardioprotective enzyme [4,46,47,48]. Therefore, studying paraoxonase enzymes and their polymorphisms in PLWH is necessary. Understanding their role and activity in this population could provide valuable insights into the mechanisms underlying metabolic alterations, oxidative stress, and their associated comorbidities, particularly in the context of prolonged antiretroviral therapy.

Therefore, we designed this case-control study to evaluate whether the frequency of PON-1 (Q192R and L55M) and PON-2 (A148G and S311C) polymorphisms, as well as PON-1 paraoxonase activity, is different among PLWH when compared to healthy individuals. Furthermore, we conducted secondary analyses to verify whether PON-1 and PON-2 polymorphisms and paraoxonase activity are associated with lipid and infection markers in PLWH using different antiretroviral therapies.

## 2. Materials and Methods

### 2.1. Study Design

**Figure 1:**
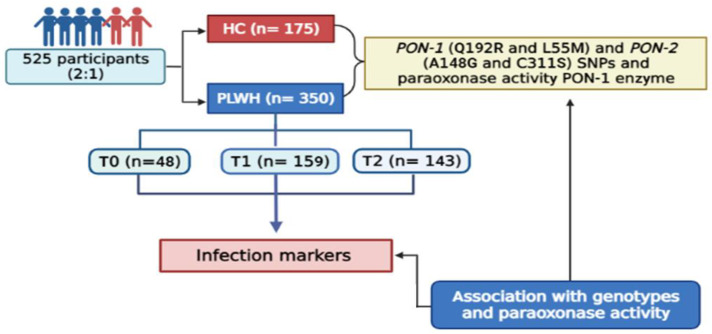
Flow-chart of this study.

This is a case-control and multicentric study comprising 525 participants with ≥18 years of age (Figure 1). The participants were divided into two groups, 350 PLWH and 175 individuals considered clinically healthy (healthy control; HC). Study participants were matched by sex at a ratio of 2:1 (male:female).

### 2.2. Study Population Criteria

#### 2.2.1. PLWH Group

The PLWH group was composed of individuals diagnosed with HIV type 1 (HIV-1) who were being monitored at the HIV-1 outpatient clinic at Casa da AIDS and the Hospital das Clínicas of the Faculty of Medicine of the University of São Paulo (HCFMUSP). All PLWH were positive for HIV type 1 (HIV-1) and each diagnosis was carried out in accordance with institutional standards recommended by the World Health Organization and Ministry of Health in Brazil [49].

PLWH were divided into three groups, T0, T1, and T2, according to their ART regimen. This classification aimed to assess whether different ART regimens influenced the paraoxonase activity of PON-1. Additionally, it is well established that ART regimens can impact lipid metabolism. The T0 group comprised 48 ART-naïve PLWH who had never undergone ART. These individuals were selected at the time of HIV-1 diagnosis. The T1 and T2 groups consisted of individuals who had been using ART for a minimum of six consecutive months.

The T1 group included 159 PLWH receiving ART based on non-nucleoside reverse transcriptase inhibitors (NNRTIs) in combination with nucleoside reverse transcriptase inhibitors (NRTIs). The treatment regimens included efavirenz (EFV) 600 mg once daily (qd) combined with azidovudine (AZT) 300 mg and lamivudine (3TC) 150 mg twice daily (bid); EFV 600 mg qd with estavudine (d4T) 40 mg and 3TC 150 mg bid; and EFV 600 mg (qd) with tenofovir (TDF) 300 mg and 3TC 150 mg (bid). Additional regimens included nevirapine (NVP) 200 mg bid combined with AZT 300 mg and 3TC 150 mg (bid); NVP 200 mg bid with d4T 40 mg and 3TC 150 mg bid; and NVP 200 mg bid with TDF 300 mg and 3TC 150 mg bid.

The T2 group consisted of 143 PLWH on ART regimens based on protease inhibitors (PIs) combined with NRTIs and/or NNRTIs. The treatment regimens also included lopinavir/ritonavir (LOP/r) 400 mg/100 mg bid combined with AZT 300 mg and 3TC 150 mg bid, as well as LOP/r 400 mg/100 mg bid with d4T 40 mg and 3TC 150 mg bid.

The selection criteria for PLWH were (I) no renal, cardiac, or hepatic function alterations and/or metabolic syndrome; (II) no co-infection, such as HPV, hepatitis B or C, or tuberculosis, in the last 6 months; (III) no use of anti-inflammatory drugs, hormones, or other medications except ART; and (IV) be positive only for HIV-1. These criteria were used to reduce the biases related to the paraoxonase activity of the PON-1 enzyme and both lipid and infection markers. The clinical and laboratory variables of infection markers were collected from medical records. For PLWH who were already under follow-up at the institution, blood samples were also collected on the day of HIV-1 infection monitoring to assess paraoxonase activity and lipid markers.

#### 2.2.2. HC Group

The HC group consisted of 175 volunteer blood donors. Individuals in the HC group were evaluated according to the guidelines set by the World Health Organization and the National Health Surveillance Agency (ANVISA) for blood donation [50,51]. This group of individuals were regular blood donors, meaning they donated blood every six months. The selection criteria were to be between 18 and 69 years of age, be in good health, weigh at least 50 kg, not use medications or have received blood transfusions, and show no clinical or analytical evidence of renal insufficiency, liver disease, neurological disease, neoplasms, dyslipidemia, hematological disorders, or chronic infections or inflammation. Furthermore, they had to be negative for clinical and laboratory evidence of transmissible infectious diseases, including hepatitis B and C, AIDS or HIV-1/2, HTLV I and II viruses, and Chagas disease. The clinical evaluation was conducted by a hematologist during donor screening. Following this, the blood donation took place and all laboratory tests, including for infectious diseases, were performed. Participants who met all the criteria described above were included in this study.

### 2.3. Sample Preparation

The venous blood samples (15 mL) were collected after 8–12 h fasting using a vacuum system with and without EDTA. Blood without EDTA was immediately centrifuged at 420× *g* for 15 min and the serum was stored at −80 °C until use. The blood collected with EDTA was used for genomic DNA extraction from leukocytes by the salting out precipitation method [52].

### 2.4. PON-1 and PON-2 Genotypes

The PON-1 polymorphisms (Q192R and L55M) were analyzed using primers designed to introduce a recognition site for the Hinf I enzyme in one allele of each PCR product. This approach enabled the simultaneous identification of both polymorphisms in a single amplification, followed by restriction enzyme analysis [53]. The Q192 and R192 alleles were determined by the presence of a 111 bp undigested fragment, along with 77 and 34 bp digested fragments. For the L55M polymorphism, L and M alleles were distinguished by a 144 bp undigested fragment and 122 and 22 bp digested fragments. Genotyping of the A148G codon of PON-2 was performed as described by Hegele et al. [54], while the S311C polymorphism was determined according to the method outlined by Motti et al. [53].

### 2.5. PON-1 Activity, Biochemical Determination and Viral Load

Here, the paraoxonase activity of PON-1 was used a marker of oxidative stress. The paraoxonase activity of PON-1 was determined according to Sentí et al. [55] and Agachan et al. [56]. The enzymatic hydrolysis of paraoxon releases *p*-nitrophenol, whose rate of formation was evaluated spectrophotometrically with absorbance readings at 405 nm at 37 °C for 10 min. PON-1 enzyme paraoxonase activity was performed in all groups.

The concentrations of serum total cholesterol (TC) as well as their fractions (HDL, LDL, and VLDL), triglycerides (TG), complete blood count/leucogram, viral load, and CD4-TL and CD8-TL lymphocyte count were measured by standard methods in a Modular 48 Analytics P-800 (Roche and Hitachi, Basel, Switzerland) automated analyzer. These clinical variables were measured in PLWH.

The HIV-1 viral load was quantified using the Nucleic Acid Sequence-Based Amplification (NASBA) kit (Organon Teknika, Boxtel, The Netherlands). The detection limit for the viral load was 50 copies/mL of HIV-1 RNA. This test was performed in both groups, PLWH and HC.

To reduce biases related to the correlation between biochemical variables, infection markers, and paraoxonase activity, all samples were collected on the same day.

### 2.6. Statistical Data Analysis

The data analysis of this research was carried out in two stages. First, we performed the comparison of demographic characteristics (sex and age), paraoxonase activity, and distribution of polymorphisms between the groups HC and PLWH. Here, the analysis performed matched sex. Second, we evaluated the difference in lipid metabolism markers and infection markers, as well as the influence of polymorphisms under these variables between the PLWH groups: T0, T1, and T2. To verify the adherence of the numerical data to a Gaussian distribution, we used the Shapiro–Wilk test, the histogram plot, and the qq-plot (quantile–quantile).

Numerical data are presented as a measure of central tendency, median with 25 and 75 percentiles, or interquartile range (IQR). Categorical variables are presented as absolute count number and frequency (n-%). The Student’s *t* test and the non-parametric Mann–Whitney u test were used to compare the quantitative variables between the groups. One-way or two-way analysis of variance with Tukey’s post-test or Kruskal–Wallis test was used for comparison among groups. The genotype frequencies of the polymorphisms were calculated using the Hardy–Weinberg Equilibrium (HWE), Chi-squared test (χ^2^), and Fisher’s exact test. Pearson (r^2^) or Spearman (ρ) correlation analysis was performed between the PON-1 activities and all other data.

Binary logistic regression analysis was used to investigate the relationship between the presence or absence of HIV-1 and the polymorphisms Q192R, L55M, A148G, and S311C. In other words, this approach assesses whether the presence of a polymorphism may be associated with the likelihood of having HIV-1. The HC and PWHL groups were defined as the dependent variable, while the polymorphisms of PON-1 and PON-2 enzymes were used as independent variables. The “wild-type” polymorphisms of each SNP were used as the reference. The forward conditional method was employed to identify significant SNPs in final multivariate analysis. The interpretation of the results was based on established mathematical parameters [57]. The results of the binary logistic regression are presented as odds ratios (ORs) with a 95% confidence interval (95% CI).

The linear regression was used to establish the relationship between lipid markers, PON-1 activity with viral load, CD4-LT, and CD8+-LT. Mathematical criteria were used to conduct the regression analysis. Briefly, (1) Linearity: the relationship between the dependent variable and each independent variable. All dependent variables (viral load, CD4-LT, and CD8+-LT) were log-transformed to reduce data dispersion and adhere to the Gaussian distribution. (2) Assessment of the independence of errors/residuals using the Durbin–Watson statistic. (3) Assessment of homoscedasticity, which should be constant, by plotting the residuals in relation to the predicted values. (4) Assessment of the Gaussian distribution of the residuals, as previously mentioned. (5) Assessment of multicollinearity using the Variance Inflation Factor (VIF) values and/or correlation matrices. VIF values above 10 were considered as the presence of multicollinearity. (6) Assessment of significant outliers using Cook’s distance statistics. Only continuous dependent variables were used in the regression analysis.

*p* ≤ 0.05 values were considered as significant. SPSS Statistics software (IBM SPSS version 30.0) was used for all analyses and the GraphPad Prism version 8.0 program was used to make the figures.

## 3. Results

### 3.1. Gender and Age of PLWH Subjects and Healthy Control Group

The population of this study consisted of 350 PLWH patients and 175 healthy individuals (Table 1). The percentage of men was higher than that of women in both groups. The mean age was higher in PLWH individuals when compared with healthy controls (*p* < 0.001).

Due to the age difference observed between the groups, we assessed whether there was a correlation between age and paraoxonase activity. No significant correlation was observed in the total population (ρ = 0.015; *p* = 0.738), nor in the HC (ρ = 0.015; *p* = 0.738), T0 (ρ = 0.016; *p* = 0.912), T1 (ρ = −0.104; *p* = 0.192), and T2 (ρ = −0.022; *p* = 0.796) groups.

### 3.2. PON-1 and PON-2 Polymorphisms

In this study, we verified whether the Q192R and L55M polymorphisms of the PON-1 gene and the A148G and S311C polymorphisms of the PON-2 gene were in Hardy–Weinberg equilibrium between the populations studied. We observed that the L55M PON-1 (*p* = 0.004) and S311C PON-2 (*p* = 0.003) polymorphisms were not in Hardy–Weinberg equilibrium (HWE). Regarding the L55M PON-1 polymorphism, we observed that there was a higher frequency of the homozygous L55L genotype in the HC group when compared to the PLWH group (49.7% and 36.9%, respectively) and, inversely, there was an increase in the frequency of the homozygous M55M in PLWH (*p* = 0.004). Interestingly, in the analysis of the genotype frequency of the S311C PON-2 genotype, we observed that the heterozygous S311C PON-2 polymorphism was more frequent in the HC group (39.2%) when compared to PLWH (24.9%) (*p* = 0.003). The other polymorphisms, C311C and S311S showed similar frequencies in both groups (Table 2). 

**Figure 2:**
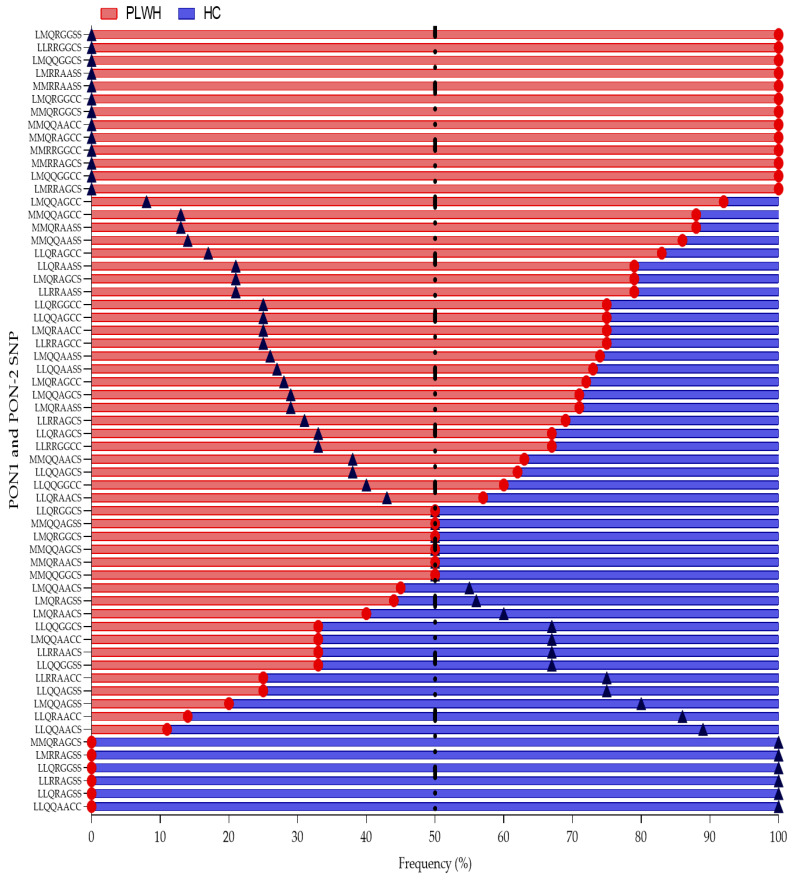
PON-1 and PON-2 genotypic frequencies. Distribution of PON-1 and PON-2 genotypic frequencies between PLWH (in red) and HC (in blue) groups. The red dots in the figure represent the genotypic frequencies for the PLWH group, while the blue triangles indicate the genotypic frequencies for the HC group. The position of the dot and triangle indicates the frequency of the genotype combination. The black dotted line marks the 50% frequency. The frequency between the groups was assessed by the Chi-squared test (*p* < 0.001)

In both populations studied, 61 combinations of complete genotypes were found (Figure 2) (*p* < 0.001). The frequency of 100% homozygous genotypes was higher in the PLWH group compared to the HC group. Of the eleven homozygous genotypes, eight were more frequent in the PLWH group: L55L-R192R-G148G-C311C (67.0%), L55L-Q192Q-A148A-S311S (73.0%), L55L-R192R-A148A-S311S (79.0%), M55M-Q192Q-A148A-S311S (86.0%), M55M-R192R-G148G-C311C (100.0%), L55L-Q192Q-G148G-C311C (60.0%), M55M-Q192Q-A148A-C311C (100.0%), and M55M-R192R-A148A-S311S (100.0%); and only three in the HC group: L55L-Q192Q-G148G-S311S (33.0%) L55L-R192R-A148A-C311C (75.0%), and L55L-Q192Q-A148A-C311C (100.0%).

In addition, the frequency of genotypes L55M-R192R-G148G-S311S, L55M-Q192Q-G148G-S311S, L55M-Q192Q-G148G-S311C, L55M-Q192R-A148A-C311C, L55M-Q192R-G148G-S311S, L55M-R192R-A148A-S311S, L55M-R192R-A148G-S311C, M55M-Q192Q-A148A-C311C, M55M-Q192R-A148G-C311C, M55M-Q192R-G418G-S311C, M55M-R192R-A148A-S311S, M55M-R192R-A148G-S311C, and M55M-R192R-G148G-C311C were 100% present only in the PLWH group.

On the other hand, the frequency of genotypes M55M-Q192R-A148G-C311S, L55M-R192R-A148G-S311S, L55L-R192R-A148G-S311S, L55L-Q192R-G148G-S311S, L55L-Q192R-A148G-S311S, and L55L-Q192Q-A148A-C311C were 100% present only in the HC group (*p* < 0.001). Through the analysis of these frequencies, it is possible to observe the higher frequency of the M allele in the PLWH population, in contrast with the higher frequency of the L allele in the HC population.

**Figure 3:**
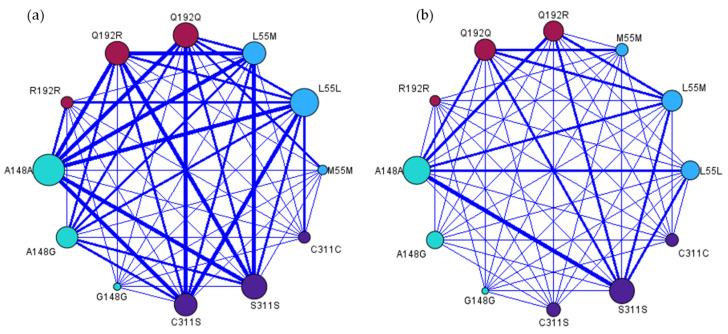
Relationship map of PON-1 and PON-2 polymorphisms in HC group (**a**) and PLWH group (**b**). The larger the circle, the greater the interaction with other polymorphisms. The blue lines represent interactions; the thicker the line, the stronger the interaction between genotypes.

The PON-1 and PON-2 genes are part of the same genetic cluster and the strength of the relationships between PON-1 and PON-2 polymorphisms in HC and PLWH was evaluated (Figure 3). It was observed that the strength of the relationships between the genetic polymorphisms was different between the two populations studied. The relationship between the polymorphisms is associated with the frequency of the genotypes in the HC and PLWH groups. In the HC group, the polymorphisms Q192Q, Q192R, L55L, and L55M of the PON-1 gene and A148A, A148G, S311S, and C311S of the PON-2 gene present greater strength of integration with the other genotypes, while in the PLWH group these genotypes present lower interaction force.

Based on the results described, the odds ratio (OR) of the susceptibility to HIV-1 infection in relation to polymorphisms of the PON-1 and PON-2 genes was calculated using univariate binary logistic regression (Table 3). First, the OR calculation was performed using the dominant or greater genotype in relation to the others. Then, individuals were grouped according to the presence of a minor allele using univariate binary logistic regression. It was observed that the presence of the homozygous L55L polymorphism of PON-1 in relation to the homozygous M55M polymorphism was associated with a 59.9% reduction in the odds of HIV-1 susceptibility (OR: 0.401; 95% CI: 0.228–0.704). Additionally, the M (LM + MM) allele was associated with 69.4% increased odds of susceptibility to HIV-1 (OR: 1.694; 95% CI: 1.173–2.446). Interestingly, the presence of the heterozygous C311S polymorphism was also associated with an increased likelihood of susceptibility to HIV-1 (OR: 2.118; 95% CI: 1.238–3.624).

Subsequently, a multivariate analysis was conducted using only the significant SNPs, L55M and C311S. In the final model, the only SNPs that remained significant were the heterozygous C311S polymorphism (β = 0.860; OR: 2.195; 95% CI: 1.276–3.775) compared to C311C and the L55L polymorphism compared to M55M (β = −0.867; OR: 0.420; 95% CI: 0.228–0.704).

### 3.3. Paraoxonase Activity

**Figure 4:**
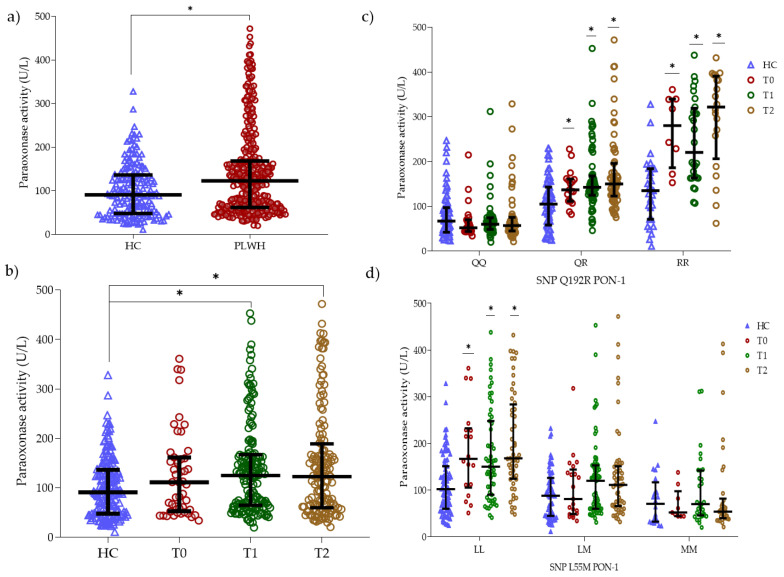
PON-1 activity. (**a**) Comparison of paraoxonase activity between the HC (in blue) and PLWH (in red) groups. (**b**) Comparison of paraoxonase activity between the HC (in blue), T0 (in red), T1 (in green), and T2 (in brown) groups. (**c**) Comparison of paraoxonase activity between the HC, T0, T1, and T2 groups by Q192R polymorphism. (**d**) Comparison of paraoxonase activity between the HC, T0, T1, and T2 groups by L55M polymorphism. The black bar represents the median and interquartile range. The HC group is represented by a triangle symbol. The PLWH group is represented by a circle. Each point represents an observation from the case series. The symbol (*) represents that the group is different from the others. The comparison between two groups was performed using the Mann–Whitney U test. The comparison between more than two groups was performed using the Kruskal–Wallis test.

The median paraoxonase activity of the PON-1 enzyme was higher in the PLWH group (123.0; IQR: 62.0–168.0) when compared to the HC group (91.0; IQR: 48.0–136.0) (*p* < 0.001) (Figure 4a). Interestingly, the comparison of the median of paraoxonase activity between the HC group and the T0 group (111.5; IQR: 54.0–161.0) was similar (*p* = 0.594). In addition, both the T1 (125.0; IQR: 65.5–166.0) and T2 (123.0; IQR: 61.0–182.0) groups showed higher paraoxonase activity when compared to the HC group (*p* = 0.002 and *p* = 0.003, respectively) (Figure 4b).

Regarding the Q192R polymorphism, the presence of the homozygous Q192Q genotype showed no difference in paraoxonase activity between the groups evaluated (*p* > 0.050). However, different paraoxonase activity was observed between the groups considering the heterozygous Q192R polymorphism, as well as in the group itself when compared to the Q192Q polymorphism. The highest activity was related to the homozygous R192R polymorphism and the activity was higher in the T0, T1, and T2 groups when compared to the HC group. Thus, the polymorphisms influence paraoxonase activity (*p* < 0.001) as well as HIV-1 infection (*p* < 0.001). Moreover, there is an interaction between the polymorphism and HIV-1 infection, regardless of the ART treatment stage, that increases the PON-1 paraoxonase activity (*p* < 0.001) (Figure 4c).

The analysis of the paraoxonase activity associated with the L55M SNP of PON-1 showed that the highest activity of the enzyme is associated with the homozygous L55L genotype when compared to the other genotypes (*p* < 0.001), being similar between the heterozygous L55M and M55M genotypes, in all groups (*p* > 0.050). Furthermore, the highest paraoxonase activity observed was in the L55L genotype in the T2, T1, and T0 groups when compared to the HC group (*p* < 0.001) (Figure 4d).

### 3.4. Cholesterol, Lipoproteins, T Lymphocytes, and Viral Load

The effects of antiretroviral therapy on cholesterol, lipoproteins, CD4 and 8 T lymphocytes, and viral load were evaluated in the three groups of PLWH (Table 4). The lowest concentration of total cholesterol (*p* = 0.050), as well as triglycerides (*p* < 0.001) and VLDL (*p* < 0.001), was observed in the T0 group when compared to the other groups. The HDL concentration was higher in the T1 group when compared to the other groups (*p* < 0.007). The LDL concentration was similar between groups (*p* = 0.106).

### 3.5. PON-1 and PON-2 Gene Polymorphisms and Paraoxonase Activity Association with Infection and Lipid Markers in PLWH

Based on previous data, we conducted secondary analyses to assess whether the presence of polymorphisms was associated with alterations in lipid metabolism markers and infection markers. All analyses are presented in the Supplementary Materials. Here we present the most relevant findings that can be used to generate hypotheses for future studies. Due to the low number of participants in each polymorphism subgroup we chose to group heterozygous individuals with smaller homozygotes. Therefore, analyses were conducted on a case-by-case basis in each antiretroviral therapy.

In the T0 group, a lower concentration of total cholesterol was observed in PLWH carriers of the R allele compared to the Q allele (*p* = 0.046) in SNP Q192R PON-1 gene. In addition, PLWH patients with R allele had a higher number of CD4-LT (*p* = 0.077), as well as a lower concentration of LDL-C (*p* = 0.065), although the statistical significance was borderline, Table S1.

In the T1 group, the highest number of CD4-TLs was observed in the patients carrying the G allele in PON-2 gene polymorphisms (A148G and G148G compared with A148A) (*p* = 0.010). As for the C311S SNP, a higher number of CD4-TLs was observed in homozygous C311C when compared to the other genotypes (*p* = 0.018). The polymorphisms in the other genes were not related with any changes in these patients (Table S2). In the T2 group, the only difference observed was in the Q192R polymorphism group of the PON-1 gene. Heterozygous (Q192R) and homozygous (R192R) had lower concentrations of LDL (*p* = 0.008) and total cholesterol (*p* = 0.052) when compared to homozygous Q192Q individuals, Table S3.

In the total PLWH group, it was observed that the highest number of CD4-LTs was associated with the R allele (59.1%) when compared with the Q allele (40.9%), while the lowest number of CD4-LTs was associated with the Q allele (51.6%) (*p* = 0.048). In the T0 group, the S311C genotype was associated with the presence of the S allele (*p* = 0.031). However, the presence of the S allele was associated with a decrease in CD4-LTs (*p* = 0.008) as well as the presence of viral load (*p* = 0.050). In the other groups, no association was observed between PON-1 and PON-2 gene polymorphisms and the decrease in CD4-LTs and detectable viral load (Table S4).

In this cohort, 96 (27.4%) PLWH had a CD4-LT count below 350 (cell/mm^3^) and 112 (32.0%) PLWH had a detectable viral load both independent of the use of ART. However, we observed a difference between the groups (T0, T1, and T2) regarding the frequency of CD4-LTs (*p* = 0.035). The low CD4-LT count in the T0 group was 11 (23.9%), that in the T1 group was 35 (22.0%), and that in the T2 group was 50 (35.0%).

The detectable viral load was 33 (68.8%) from the T0 group, 28 (17.6%) from the T1 group, and 51 (35.7%) from the T2 group (*p* < 0.001). As for the paraoxonase activity of the PON-1 enzyme, no difference was observed between PLWH for the viral load (*p* = 0.254). On the other hand, paraoxonase activity was lower in PLWH with a low CD4-LT count when compared to PLWH with a high CD4-LT (*p* = 0.010).

To assess the influence of lipids on the number of CD4-LTs and CD8-LTs, univariate linear regression analyses were performed with each of the biochemical parameters studied (Table S5). In the T0 group, serum HDL concentration was associated with the amount of CD8-LTs, which was about 31% (R^2^ = 0.317; *p* = 0.008). Similarly, in the T2 group, total cholesterol concentration was associated with an increase in CD4-LTs (β = 0.008; R^2^ = 0.115; *p* = 0.030). In this study, it was not possible to perform multivariate regression analysis.

## 4. Discussion

In this study, we evaluated paraoxonase 1 and 2 polymorphisms, as well as PON-1 paraoxonase activity, in PLWH and healthy individuals. To determine whether polymorphisms in the PON-1 and PON-2 genes, along with paraoxonase activity, are associated with HIV-1 infection in PLWH, we conducted a sex-matched case-control study. This is the first study to assess these polymorphisms together in PLWH. Among the four single nucleotide polymorphisms analyzed, we found that the L55M PON-1 and C311S PON-2 polymorphisms deviated from a Hardy–Weinberg equilibrium between the PLWH and HC groups. The PON-1 and PON-2 genes are part of the same genetic cluster on human chromosome 7, and their respective polymorphisms—L55M, Q192R, A148G, and C311S—have been linked to the development of various metabolic disorders, particularly those related to lipid metabolism and cardiovascular diseases [27,58,59,60].

A predominance of genotypes associated with the L55M and M55M PON-1 polymorphisms was observed in the PLWH group, whereas the L55L PON-1 genotype was predominantly found in the HC group. Additionally, the M allele was more frequent in PLWH. In the odds ratio analysis, individuals homozygous for L55L exhibited an approximately 60% protective effect against HIV-1. This is the first time that the L55M PON-1 polymorphism has been associated with HIV-1 infection. Although this finding needs to be confirmed in further studies, the presence of the M variant (LM and/or MM), compared to the L allele (LL), has been linked to the development of several diseases [61], including increased susceptibility to non-alcoholic fatty liver disease [62], cancer [6,63], and other conditions [14,64,65,66].

Regarding the S311C polymorphism of PON-2, the homozygous C311C genotype was more prevalent in the PLWH group compared to the HC group. The PON-2 enzyme is located on the plasma membrane, and in vitro studies have shown that its lactonase activity and pro-apoptotic function contribute to defense against Gram-negative bacterial infections by regulating biofilm formation and the production of virulence factors [23]. Additionally, in vitro evidence suggests that the A148G and S311C polymorphisms may influence post-translational modifications, indicating a direct modulatory effect of these SNPs. Mutant C311C proteins exhibit a 30-fold reduction in esterase catalytic activity [67].

The Q192R and A148G polymorphisms exhibited similar frequencies between the groups. However, differences were observed in the distribution of complete genotypes and the interaction strengths of these genotypes within the evaluated groups. Association studies between PON-1 and PON-2 genetic cluster polymorphisms are scarce. We found 61 different combinations between the genotypes Q192R, L55M, A148G, and S311C, and these combinations were different between PLWH and HC, showing that the difference may be associated with HIV-1 infection. However, these results must be proven in future studies.

Although the physiological substrates of PON-1 remain unknown, in vitro studies have described three main classes of substrates for PON-1 that may be related to its enzymatic antioxidant activity in humans: the hydrolysis of lactones through lactonase activity, the hydrolysis of aryl esters through arylesterase activity, and the hydrolysis of organophosphates, primarily paraoxon, through paraoxonase activity [68]. The paraoxonase activity of the PON-1 enzyme is one of the most extensively studied in diseases associated with increased oxidative stress, inflammation, and lipid metabolism disorder [69,70,71,72].

Here, the paraoxonase activity was higher in PLWH when compared with healthy individuals. This result suggests that the increased paraoxonase activity of PON-1 in PLWH is a metabolic antioxidant response to the oxidative stress caused by HIV-1 infection and prolonged ART use. In fact, in PLWH with an undetectable viral load, prolonged ART use for approximately 10 years has been shown to result in a pronounced redox imbalance and triple ART therapies have been associated with increased oxidative stress. Furthermore, the use of protease inhibitors has been linked to a higher production of reactive oxygen metabolites [73].

PLWH using both NRTI- and PI-based ART presented increased paraoxonase activity, which is inconsistent with findings from previous studies on the lactonase, arilesterase, and paraoxonase activities of the PON-1 in this population [74,75,76]. These divergent results may be related to the mechanism of action of the PON-1 enzyme in detoxifying different xenobiotics, as well as to the use of distinct substrates to measure PON-1 activity [76,77,78,79,80]. Furthermore, the median paraoxonase activity was similar between the HC and ART-naïve group. These findings are particularly interesting as they highlight the influence of antiretroviral treatment on paraoxonase activity. It is likely that, at the onset of HIV-1 infection, paraoxonase activity related to oxidative stress remains unchanged, whereas prolonged antiretroviral therapy contributes to increased oxidative stress. As a compensatory mechanism, these combined factors may enhance PON-1 paraoxonase activity in an attempt to maintain oxidative homeostasis [77,78,79,80].

Conversely, a separate study revealed lower concentrations of paraoxonase PON-1, PON-3, and HDL in the PLWH cohort when compared to the control group. Furthermore, paraoxonase activity was significantly elevated in the control group relative to both the ART-naïve group and those receiving NNRTI-based ART, regardless of whether they had a detectable viral load or were treated with protease inhibitors [81]. Prolonged use of NNRTIs was too associated with a reduction in serum PON-3 concentrations [82]. All these findings, including those observed in our study, suggest that antiretroviral treatments may induce oxidative stress through the downregulation of the paraoxonase family. The increase in paraoxonase activity observed here may provide important insights into the development of comorbidities in PLWH.

The PON-1 enzyme exhibits anti-inflammatory and antioxidant functions, which are believed, in part, to be linked to its relationship with HDL, as well as to be due to its affinity for various substrates [83,84]. PON-1 circulates in the human body bound to HDL. HDL contains other enzymes in its structure, such as Apo-A1. It is suggested that both HDL and Apo-A1 are associated with PON-1 activities and that reduced enzyme activity is associated with the development of diseases or a pro-disease status [85]. Due to these characteristics related to metabolism, paraoxonase activity is known to be cardioprotective, primarily with anti-atherogenic functions. However, it is important to note that this function is not exclusive to PON-1, as it is shared with HDL [86,87].

The subjects in the T0 group had low concentrations of serum total cholesterol, VLDL-C, and triglycerides when compared to T1 and T2 groups. These results demonstrate that at the onset of HIV-1 infection, there are no evident changes in lipid metabolism. In this study, the ART in the T1 group was a combination of efavirenz, zidovudine, lamivudine, stavudine, tenofovir, and nevirapine. In an in vitro model of liver cells, Marinho and colleagues [88] reported that both nevirapine and its metabolite 12-hydroxy-nevirapine increased paraoxonase activity. These results were confirmed in a human study, where nevirapine and its metabolite were associated with increased HDL-C and paraoxonase activity [89]. In a study with rats that received the protease inhibitors ritonavir, atazanavir, and saquinavir for four weeks, paraoxonase activity was decreased, suggesting that these medications may lead to an exhaustion of PON-1 paraoxonase activity [90]. Furthermore, the prolonged use of protease inhibitors, particularly lopinavir/ritonavir, is associated with the development of dyslipidemia, characterized by increase in both LDL-C and cardiovascular risk [91,92].

Here, no difference in LDL-C concentration was observed between the T0, T1, and T2 groups. However, triglyceride and VLDL-C concentrations were higher in the T2 group, which used protease inhibitors. Although paraoxonase activity was increased in both the NNRTI-based and protease inhibitor groups compared to healthy controls, our study did not demonstrate any strong correlation between paraoxonase activity, infection, and lipid markers in any of the groups analyzed. Thus, based on all the observed results related to paraoxonase activity in the different groups of PLWH evaluated, we can observe that, while there is no increase in oxidative stress in the T0 group, the paraoxonase activity under PON-1 is similar to that of healthy controls. On the other hand, the increase in paraoxonase activity in the groups with different ARTs can be considered an indication of oxidative stress in this population.

Indeed, environmental and molecular–genetic factors alter the enzyme’s function regarding the hydrolysis of substrates, which favors an increase in oxidative stress [14,15,66]. In addition, the highest paraoxonase activity was associated with the Q192R and R192R genotypes. In PLWH, the enzyme activity in these genotypes was approximately 1.5 times higher than in healthy individuals. Interestingly, the highest CD4-TL cell count was observed in individuals with the R allele. On the other hand, the lowest enzyme activity was found in genotypes Q192Q, L55M, and M55M. Both genotypes are known to be slow metabolizers, especially in individuals carrying the M and Q alleles [93]. Through these results, it is possible to observe the joint influence of antiretroviral treatment and the presence of L55M and Q192R polymorphisms on the paraoxonase activity of PON-1.

Our results of association between paraoxonase activity and imbalance in the presence of polymorphisms in the populations studied can be used to infer that HIV-1-infected individuals are likely to have an imbalance in their antioxidant status associated with the gene cluster of the paraoxonase family. In addition, these characteristics may contribute to viral infection, in part, by maintaining an environment conducive to the development of the virus disease progression [94,95,96,97,98]. The mean age of the PLWH group was higher compared to the HC group. However, no correlation was observed here between participant age and PON-1 paraoxonase activity. The relationship between age and PON-1 paraoxonase activity remains controversial [99,100]. However, it is possible that paraoxonase activity decreases with aging [101].

Systemic alterations in lipid metabolism, such as reduced HDL cholesterol levels and changes in triglycerides, were observed, indicating that the impact of HIV-1 and ART extends beyond viral control, encompassing potential metabolic and cardiovascular risks. These findings emphasize the need for personalized therapeutic strategies for PLWH, considering both genetic and metabolic factors associated with prolonged ART use. Additionally, monitoring paraoxonase activity and lipid profiles based on polymorphisms may be useful for preventing related complications in PLWH. Prolonged ART use in PLWH can lead to lipodystrophy, bone mass loss, diarrhea, lean tissue depletion, increased lipids, insulin resistance, diabetes, atherosclerosis, and cardiovascular diseases, all of which contribute to an elevated risk of mortality [102,103,104].

This study has some limitations. First, sample size biases may have affected the genotypic frequencies observed. Additionally, the Hardy–Weinberg imbalance for the L55M and S311C polymorphisms could be due to a lack of genetic representativeness in the populations studied. The cross-sectional design also limits the ability to establish causal relationships between the PON-1 and PON-2 gene polymorphisms, ART, lipid profile alterations, and paraoxonase activity. While we controlled for sex in our case-control design, other factors such as environmental exposure, lifestyle, demographics, ethnicity, and epigenetics, which can influence oxidative stress and lipid changes, were not considered.

Future studies should adopt a longitudinal approach to better understand the causal links between genetic polymorphisms, ART, and metabolic changes, with a focus on sample diversification. Additionally, it is important to explore the molecular and pathophysiological mechanisms underlying HIV-1 infection and the role of PON-1 and PON-2 polymorphisms, along with their enzyme activities, to reduce oxidative stress in PLWH. This could involve introducing potential antioxidant compounds that could enhance PON-1 function.

In conclusion, our data show that the frequency of the M55M genotype and/or the M allele is higher in PLWH. Specifically, the M allele may be linked to the susceptibility to HIV-1, while the L55L genotype may offer partial protection against infection. In addition, paraoxonase activity in PLWH was higher in groups using ART, suggesting that prolonged use of ART may positively influence the antioxidant activity of this enzyme in response to increased oxidative stress caused by both infection and treatment and that polymorphisms such as L55M and Q192R in the PON-1 gene are associated with variations in enzyme activity.

## Figures and Tables

**Table 1:** Gender and age of patients with HIV-1 and healthy controls.

Gender	Groups (*n* = 525)		
	HC (*n* = 175)	PLWH (*n* = 350)	*p* Value ^(1)^
*Female*	43 (24.6%)	86 (24.6%)	1.000
*Male*	132 (75.4%)	264 (75. 4%)	1.000
Age (years)	37.4 ± 9.0	41.5 ± 10.9	<0.001

^(1)^ Comparison between groups using Chi-squared test (χ^2^) and Student’s *t* test. Abbreviation: HC = healthy control; PLWH = people living with HIV-1; *n* = number of individuals; % = percentage.

**Table 2:** Distribution of genotypes and allelic frequency of *PON-1* Q192R and L55M polymorphisms and *PON-2* A148G and C311S polymorphisms in HC and PLWH.

Gene-Polymorphism		Group (*n* = 525)				
		HC (*n* = 175)		PLWH (*n* = 350)		*p* Value ^(1)^
PON-1 Q192R	QQ	75	42.9%	152	43.6%	0.970
	QR	71	40.6%	142	40.7%	
	RR	29	16.6%	55	15.8%	
	Q allele	221	63.1%	446	63.9%	
	R allele	129	36.9%	252	36.1%	
PON-1 L55M	LL	87	49.7%	129	36.9%	0.004
	LM	68	38.9%	147	42.0%	
	MM	20	11.4%	74	21.1%	
	L allele	242	69.1%	405	57.9%	
	M allele	108	30.9%	295	42.1%	
PON-2 A148G	AA	99	56.9%	209	59.7%	0.811
	AG	63	36.2%	117	33.4%	
	GG	12	6.9%	24	6.9%	
	A allele	262	75.0%	535	76.4%	
	G allele	87	25.0%	165	23.6%	
PON-2 S311C	SS	76	44.4%	186	53.1%	0.003
	CS	67	39.2%	87	24.9%	
	CC	28	16.4%	77	22.0%	
	C allele	123	36.0%	241	34.4%	
	S allele	219	64.0%	459	65.6%	

Results are expressed as *n* (%); ^(1)^ Comparison between groups using Chi-squared test (χ^2^) or Fisher’s exact test. Abbreviation: HC = healthy control; PLWH = people living with HIV-1; *n* = number of individuals; % = percentage.

**Table 3:** Univariate binary logistic regression between the presence of polymorphisms PON-1 and PON-2 and susceptibility to HIV-1.

Polymorphism	β	SE	Wald	*p* Value	Exp(β)	Exp(β) CI 95%	
						Lower	Upper
Q192Q			0.032	0.984			
Q192R	0.013	0.203	0.004	0.948	1.013	0.681	0.507
R192R	0.048	0.269	0.032	0.857	1.050	0.620	1.777
Constant	−0.706	0.141	25.059	<0.001	0.493		
R allele	0.023	0.187	0.016	0.901	1.024	0.710	1.477
Constant	−0.730	0.308	50.622	0.018	0.482		
L55L			10.848	0.004			
L55M	−0.377	0.202	30.488	0.062	0.686	0.462	1.019
M55M	−0.914	0.288	10.104	0.001	0.401	0.228	0.704
Constant	−0.394	0.139	80.062	0.005	0.674		
M allele	0.527	0.187	7.902	0.005	1.694	1.173	2.446
Constant	−0.921	0.126	53.38	<0.001	0.398		
A148A			0.418	0.811			
A148G	0.128	0.198	0.418	0.518	1.137	0.771	1.677
G148G	0.054	0.374	0.021	0.885	1.056	0.507	2.197
Constant	−0.747	0.122	37.508	<0.001	0.474		
G allele	0.116	0.188	0.381	0.537	1.123	0.777	1.623
Constant	−0.747	0.122	37.508	<0.001	0.474		
C311C			11.332	0.003			
C311S	0.750	0.274	7.496	0.006	2.118	1.238	3.624
S311S	0.117	0.259	0.202	0.653	1.124	0.676	1.868
Constant	−1.012	0.221	21.013	<0.001	0.364		
S allele	0.365	0.244	2.244	0.134	1.440	0.894	2.322
Constant	−1.012	0.221	21.013	<0.001	0.364		

Binary logistic regression parameters and interpretation: The β (coefficient) indicates the direction and magnitude of the polymorphism’s effect, with positive values increasing the chances of the event and negative values decreasing them. SE (standard error) reflects the precision of the coefficient. Wald assesses the relevance of the variable, with larger values indicating greater importance. *p*-value indicates statistical significance, being significant when less than 0.050. Exp(β) (odds ratio) shows how the chances of the event change with each unit of polymorphism change, with values > 1 increasing and <1 decreasing the chances. Exp(β) CI 95% shows the confidence interval for the OR, indicating the range of values with 95% certainty. The constant represents the probability of the event in the absence of independent variables; if negative, the chances are low, and if positive, high.

**Table 4:** Cholesterol, lipoproteins, CD4 and CD8 T lymphocytes, and viral load in PLWH.

	PLWH (*n* = 350)									
	*T0 (n = 48)*			*T1 (n = 159)*				*T2 (n = 143)*		
	*Median*	*P25*	*P75*	*Median*	*P25*	*P75*	*Median*	*P25*	*P75*	*p Value*
*CD4-TLs* (mil/mm^3^)	462.0	351.0	629.0	534.0	376.0	704.0	455.0	269.0	632.0	0.004
*CD8-TLs* (mil/mm^3^)	1001.5	746.0	1289.0	796.0	604.0	1042.0	952.0	726.0	1295.0	<0.001
*Viral load (Log)*	4.4	3.7	5.0	3.9	3.1	4.6	4.5	3.8	5.1	0.048
*Cholesterol* (mg/dL)	173.0	150.0	192.0	187.0	160.0	214.0	172.0	151.0	210.0	0.050
*HDL-C* (mg/dL)	41.5	38.0	58.0	50.0	39.0	62.0	43.0	35.0	53.0	0.007
*LDL-C* (mg/dL)	94.5	67.0	119.0	105.0	81.0	131.0	98.0	70.0	121.0	0.106
*VLDL-C* (mg/dL)	24.5	18.0	36.0	25.0	18.0	40.0	34.0	23.0	44.0	<0.001
*TG* (mg/dL)	124.5	91.0	190.0	131.0	92.0	220.0	185.0	124.0	247.0	<0.001

Comparison among the groups using Kruskal–Wallis test. Abbreviations: PLWH = people living with HIV-1; *n* = number of individuals; TL: T lymphocyte; HDL-C: high-density lipoprotein cholesterol; LDL-C: low-density lipoprotein cholesterol; TG: triglycerides; VLDL-C: very low-density lipoprotein cholesterol. Data are presented as median and 25 percentile (P25) and 75 percentile (P75).

## Data Availability

The data generated in this research can be made available upon consultation with the main researcher, S.P.B. The data will be made available anonymously. Data on genotyping and PON-1 enzyme activity will be available. Data from medical records of study participants cannot be made available.

## Supplementary Materials

The following supporting information can be downloaded at: https://www.mdpi.com/article/10.3390/antiox14020209/s1, Table S1: Comparison of biochemical variables between the SNPs in the T0 group (*n* = 48); Table S2: Comparison of biochemical variables between the SNPs in the T1 group (*n* = 159); Table S3: Comparison of biochemical variables between the SNPs in the T2 group (*n* = 143); Table S4: Association between SNPs of PON-1 e PON-2 genes with CD4-LT number 19 and viral load in PLWH; Table S5: Association between biochemical variables with viral load, CD4-TLs, and 24 CD8-TLs in the PLWH group.
