# Supplementary material for: PON-1 and PON-2 Polymorphisms and PON-1 Paraoxonase Activity in People Living with HIV-1
Source: Antioxidants (Basel). 2025 Feb 12;14(2):209. doi: 10.3390/antiox14020209 (PMC11851513; doi:10.3390/antiox14020209)
Supplement: Supplementary file 1 [file antioxidants-14-00209-s001.zip › antioxidants-3332912-supplementary.pdf]

Supplementary materials

Table S1. Comparison of biochemical variables between the SNPs in the T0 group (n=48).

**Variables**	**PON-1 Q192R**						
	**Q (n=23)**			**R (n=25)**			
	Median	P25	P75	Median	P25	P75	**P value**
Paraoxonase (U/L)	52.0	44.0	70.0	158.0	133.0	228.0	<0.001
CD4-TL(mil/mm^3)	406.0	321.0	533.0	540.0	389.0	695.0	0.077
CD8-TL(mil/mm^3)	1074.0	704.0	1383.0	987.0	746.0	1289.0	0.410
Viral load (Log)	4.5	4.2	5.0	4.2	3.6	5.0	0.326
Cholesterol (mg/dL)	186.0	173.0	199.0	163.0	132.0	181.0	0.046
HDL-C (mg/dL)	49.0	39.0	58.0	40.0	34.0	57.0	0.363
LDL-C (mg/dL)	110.0	92.0	124.0	77.0	61.0	112.0	0.065
VLDL-C (mg/dL)	23.0	15.0	29.0	29.0	21.0	38.0	0.170
TG (mg/dL)	116.0	77.0	147.0	145.0	107.0	190.0	0.281

**Variables**	**PON-1 L55M**						
	**L (n=18)**			**M (n=30)**			
	Median	P25	P75	Median	P25	P75	**P value**
Paraoxonase(U/L)	167.0	111.0	229.0	66.5	48.0	134.0	<0.001
CD4-TL(mil/mm^3)	488.0	430.0	556.0	410.0	336.0	675.0	0.847
CD8-TL(mil/mm^3)	925.0	746.0	1226.0	1074.0	770.0	1383.0	0.241
Viral load (Log)	4.5	4.1	5.0	4.2	3.7	5.0	0.326
Cholesterol (mg/dL)	162.0	150.0	181.0	177.0	157.0	197.0	0.258
HDL-C (mg/dL)	40.5	39.5	57.5	43.5	34.0	58.0	0.573
LDL-C (mg/dL)	90.5	65.0	115.5	95.5	77.0	124.0	0.545
VLDL-C (mg/dL)	22.5	17.0	28.5	29.0	18.0	38.0	0.267
TG (mg/dL)	113.5	84.0	144.5	146.0	91.0	191.0	0.232

**Variables**	**PON-2 A148G**						
	**A (n=28)**			**G (n=20)**			
	Median	P25	P75	Median	P25	P75	**P value**
Paraoxonase(U/L)	97.5	48.0	152.0	130.5	71.0	173.5	0.132
CD4-TL(mil/mm^3)	442.0	321.0	629.0	488.0	389.0	695.0	0.384
CD8-TL(mil/mm^3)	999.0	770.0	1336.0	1004.0	626.0	1289.0	0.867
Viral load (Log)	4.5	3.8	5.1	4.3	3.7	4.9	0.683
Cholesterol (mg/dL)	174.5	162.0	189.0	157.0	136.0	199.0	0.489
HDL-C (mg/dL)	42.0	39.0	52.0	40.0	31.0	64.0	0.967
LDL-C (mg/dL)	103.0	77.0	119.0	87.0	61.0	110.0	0.363
VLDL-C (mg/dL)	24.0	18.0	32.0	27.0	21.0	38.0	0.742

 https://doi.org/10.3390/antiox14020209

TG (mg/dL)	120.0	91.0	162.0	134.0	107.0	190.0	0.869
	**PON-2 C311S**						
**Variables**	**C (n=11)**			**S (n=37)**			
	Median	P25	P75	Median	P25	P75	**P value**
Paraoxonase(U/L)	134.0	75.0	172.0	111.0	50.0	155.0	0.297
CD4-TL(mil/mm³)	491.0	456.0	695.0	430.0	321.0	629.0	0.094
CD8-TL(mil/mm³)	886.0	605.0	1457.0	1004.0	797.0	1250.0	0.703
Viral load (Log)	4.3	3.7	4.6	4.5	3.7	5.1	0.630
Cholesterol (mg/dL)	173.0	136.0	199.0	170.0	157.0	189.0	0.848
HDL-C (mg/dL)	40.0	34.0	64.0	42.0	39.0	53.0	0.965
LDL-C (mg/dL)	87.0	61.0	110.0	99.0	77.0	119.0	0.563
VLDL-C (mg/dL)	27.0	23.0	38.0	24.0	18.0	32.0	0.657
TG (mg/dL)	134.0	116.0	190.0	120.0	91.0	162.0	0.756

Comparison between the groups using Mann-Whitney u test; Abbreviation: n= number of individuals; TL: T lymphocyte; HDL-C: high density lipoprotein-cholesterol; LDL-C: low density lipoprotein-cholesterol; TG: triglycerides; VLDL-C: very low-density lipoprotein cholesterol; Data are presented as median and 25 percentile (P25) and 75 percentile (P75).

Table S2. Comparison of biochemical variables between the SNPs in the T1 group (n=159).

	**PON-1 Q192R**						
**Variables**	**Q (n=64)**			**R (n=78)**			
	Median	P25	P75	Median	P25	P75	**P value**
Paraoxonase(U/L)	60.0	49.0	74.0	153.5	129.0	233.0	<0.001
CD4-TL(mil/mm³)	504.0	336.0	701.0	572.0	409.0	704.0	0.296
CD8-TL(mil/mm³)	840.0	601.0	1028.0	780.5	615.0	1045.0	0.835
Viral load (Log)	3.8	3.1	5.0	3.9	3.1	4.4	0.802
Cholesterol (mg/dL)	193.0	162.0	217.0	182.0	158.0	212.0	0.246
HDL-C (mg/dL)	51.0	39.0	60.0	49.0	40.0	62.0	0.816
LDL-C (mg/dL)	107.5	85.0	135.0	102.0	80.0	126.0	0.164
VLDL-C (mg/dL)	28.5	19.0	40.0	24.0	18.0	34.0	0.308
TG (mg/dL)	150.5	95.0	233.0	120.0	90.0	215.0	0.193
	**PON-1 L55M**						
**Variables**	**L (n=52)**			**M (n=91)**			
	Median	P25	P75	Median	P25	P75	**P value**
Paraoxonase(U/L)	150.0	90.0	248.0	85.0	57.0	149.5	**<0.001**
CD4-TL(mil/mm³)	587.0	362.0	761.0	530.0	381.0	700.5	0.470
CD8-TL(mil/mm³)	829.0	610.0	1063.0	769.5	600.0	1012.5	0.370
Viral load (Log)	4.0	3.7	4.5	3.5	3.1	4.8	0.410
Cholesterol (mg/dL)	186.0	158.0	216.0	188.0	160.0	212.0	0.650
HDL-C (mg/dL)	47.0	38.0	53.9	51.0	40.0	62.0	0.133
LDL-C (mg/dL)	106.0	80.0	126.0	103.5	82.0	133.0	0.485
VLDL-C (mg/dL)	27.0	18.6	41.0	24.0	18.0	36.8	0.242

TG (mg/dL)	151.0	98.0	250.0	123.0	89.0	203.0	0.201

**Variables**	**PON-2 A148G**						
	**A (n=101)**			**G (n=58)**			
	Median	P25	P75	Median	P25	P75	**P value**
Paraoxonase(U/L)	126.0	60.0	167.0	121.5	70.0	163.0	0.869
CD4-TL(mil/mm³)	502.0	355.0	658.0	617.5	433.0	831.0	0.010
CD8-TL(mil/mm³)	770.0	608.0	993.0	862.0	604.0	1102.0	0.214
Viral load (Log)	3.7	3.1	4.6	4.0	3.4	4.5	0.672
Cholesterol (mg/dL)	188.0	160.0	214.0	184.0	157.0	214.0	0.699
HDL-C (mg/dL)	51.0	42.0	64.0	47.0	37.0	54.0	0.056
LDL-C (mg/dL)	100.5	81.0	127.0	109.0	84.0	131.0	0.637
VLDL-C (mg/dL)	25.0	18.0	40.5	27.0	17.0	34.0	0.821
TG (mg/dL)	129.0	92.5	215.5	136.0	85.0	232.0	0.889

**Variables**	**PON-2 C311S**						
	**C (n=31)**			**S (n=112)**			
	Median	P25	P75	Median	P25	P75	**P value**
Paraoxonase(U/L)	129.0	62.0	163.0	124.5	65.5	168.0	0.821
CD4-TL(mil/mm³)	684.0	433.0	851.0	528.5	353.0	673.0	0.018
CD8-TL(mil/mm³)	937.0	610.0	1128.0	774.5	599.5	1023.5	0.161
Viral load (Log)	4.0	3.5	4.2	3.7	3.1	4.8	0.816
Cholesterol (mg/dL)	190.0	166.0	207.0	185.0	160.0	217.0	0.982
HDL-C (mg/dL)	47.0	36.0	53.0	50.5	40.0	63.0	0.065
LDL-C (mg/dL)	110.0	91.0	129.0	102.5	81.0	131.0	0.637
VLDL-C (mg/dL)	30.0	17.0	41.0	25.0	18.0	37.0	0.547
TG (mg/dL)	164.0	83.0	259.0	127.0	92.0	214.0	0.386

Comparison between the groups using Mann-Whitney u test; Abbreviation: n= number of individuals; TL: T lymphocyte; HDL-C: high density lipoprotein-cholesterol; LDL-C: low density lipoprotein-cholesterol; TG: triglycerides; VLDL-C: very low-density lipoprotein cholesterol; Data are presented as median and 25 percentile (P25) and 75 percentile (P75).

Table S3. Comparison of biochemical variables between the SNPs in the T2 group (n=143).

**Variables**	**PON-1 Q192R**						
	Q (n=64)			R (n=79)			
	Median	P25	P75	Median	P25	P75	**P value**
Paraoxonase(U/L)	57.0	45.0	75.0	162.0	125.0	289.0	<0.001
CD4-TL(mil/mm³)	462.0	272.5	657.5	453.0	264.0	601.0	0.643
CD8-TL(mil/mm³)	931.5	804.0	1300.5	995.0	684.0	1253.0	0.784
Viral load (Log)	4.4	3.8	4.8	4.6	3.9	5.1	0.196
Cholesterol (mg/dL)	185.0	158.0	221.0	167.5	147.0	199.0	0.052
HDL-C (mg/dL)	43.0	35.0	51.0	43.5	35.0	59.0	0.775
LDL-C (mg/dL)	105.0	85.0	129.0	89.0	67.0	112.0	0.008
VLDL-C (mg/dL)	33.2	23.0	49.0	34.5	24.0	43.0	0.765
TG (mg/dL)	195.0	127.0	319.0	177.0	121.0	220.0	0.153

Variables	PON-1 L55M						
	**L (n=52)**			**M (n=91)**			
	Median	P25	P75	Median	P25	P75	**P value**
Paraoxonase(U/L)	168.0	125.5	280.0	76.0	52.0	142.0	<0.001
CD4-TL(mil/mm³)	546.0	304.0	675.5	416.0	249.0	577.0	0.083
CD8-TL(mil/mm³)	946.0	661.5	1314.0	952.0	799.0	1268.0	0.786
Viral load (Log)	4.4	3.8	4.9	4.7	3.9	5.1	0.615
Cholesterol (mg/dL)	174.0	149.0	199.0	171.5	152.0	211.0	0.707
HDL-C (mg/dL)	45.0	36.0	59.0	42.0	34.0	52.0	0.493
LDL-C (mg/dL)	98.0	70.0	119.0	97.5	71.0	121.0	0.819
VLDL-C (mg/dL)	32.0	23.0	42.0	35.0	25.0	44.0	0.407
TG (mg/dL)	170.0	117.0	233.0	189.0	126.0	254.0	0.419
**Variables**	**PON-2 A148G**						
	**A (n=80)**			**G (n=63)**			
	Median	P25	P75	Median	P25	P75	**P value**
Paraoxonase(U/L)	111.0	54.5	179.0	131.0	66.0	189.0	0.132
CD4-TL(mil/mm³)	453.0	274.0	579.5	508.0	264.0	673.0	0.351
CD8-TL(mil/mm³)	1010.0	733.0	1388.5	904.0	726.0	1249.0	0.319
Viral load (Log)	4.6	3.8	5.1	4.4	4.2	5.1	0.962
Cholesterol (mg/dL)	171.5	151.5	205.5	181.0	145.0	214.0	0.708
HDL-C (mg/dL)	43.0	34.5	54.0	43.0	35.0	53.0	0.984
LDL-C (mg/dL)	100.0	72.0	117.0	92.0	70.0	129.0	0.687
VLDL-C (mg/dL)	33.0	22.0	44.0	35.0	25.0	43.0	0.626
TG (mg/dL)	183.5	110.5	255.5	185.0	125.0	233.0	0.920
**Variables**	**PON-2 S311C**						
	**C (n=31)**			**S (n=112)**			
	Median	P25	P75	Median	P25	P75	**P value**
Paraoxonase(U/L)	67.0	226.0	113.5	58.5	171.5	67.0	0.296
CD4-TL(mil/mm³)	264.0	706.0	453.0	274.0	584.5	264.0	0.370
CD8-TL(mil/mm³)	820.0	1192.0	937.0	709.0	1351.5	820.0	0.969
Viral load (Log)	4.3	5.1	4.6	3.8	5.0	4.3	0.868
Cholesterol (mg/dL)	154.0	214.0	170.0	151.0	204.0	154.0	0.339
HDL-C (mg/dL)	37.0	53.0	43.0	35.0	53.0	37.0	0.711
LDL-C (mg/dL)	70.0	130.0	97.5	71.0	118.0	70.0	0.548
VLDL-C (mg/dL)	26.0	46.0	33.0	22.0	42.0	26.0	0.186
TG (mg/dL)	130.0	233.0	177.0	117.0	250.0	130.0	0.387

Comparison between the groups using Mann-Whitney u test; Abbreviation: n= number of individuals; TL: T lymphocyte; HDL-C: high density lipoprotein-cholesterol; LDL-C: low density lipoprotein-cholesterol; TG: triglycerides; VLDL-C: very low-density lipoprotein cholesterol; Data are presented as median and 25 percentile (P25) and 75 percentile (P75).

Table S4. Association between SNPs of PON-1 e PON-2 genes with CD4-LT number and viral load in PLWH.

Groups	SNP	Allele	CD4-TL				*P*	Viral load				*P*
			Low		High			Yes		No		
			N	%	N	%	*value*	N	%	N	%	*value*
PLWH (n=350)	Q192R PON-1	Q	49	51.6	103	40.9	**0.048**	49	44.1	103	43.3	0.879
		R	46	48.4	**149**	**59.1**		62	55.9	135	56.7	
	L55M PON-1	L	32	33.3	96	38.1	0.408	39	34.8	90	37.8	0.587
		M	64	66.7	156	61.9		73	65.2	148	62.2	
	A148G PON-2	A	62	64.6	146	57.9	0.258	66	58.9	143	60.1	0.837
		G	34	35.4	106	42.1		46	41.1	95	39.9	
	C311S PON-2	C	16	16.7	61	24.2	0.083	25	22.3	52	21.8	0.921
		S	80	83.3	191	75.8		87	77.7	186	78.2	
T0 (n=48)	Q192R PON-1	Q	8	72.7	15	42.9	0.083	16	48.5	7	46.7	0.907
		R	3	27.3	20	57.1		17	51.5	8	53.3	
	L55M PON-1	L	3	27.3	14	40.0	0.349	12	36.4	6	40.0	0.810
		M	8	72.7	21	60.0		21	63.6	9	60.0	
	A148G PON-2	A	9	81.8	18	51.4	0.073	17	51.5	11	73.3	0.134
		G	2	18.2	17	48.6		16	48.5	4	26.7	
	C311S PON-2	C	0	0.0	11	31.4	**0.008**	10	30.3	1	6.7	**0.050**
		S	11	100.0	24	68.6		23	69.7	14	93.3	
T1 (n=159)	Q192R PON-1	Q	18	51.4	47	37.9	0.153	12	42.9	53	40.5	0.815
		R	17	48.6	77	62.1		16	57.1	78	59.5	
	L55M PON-1	L	14	40.0	45	36.3	0.689	9	32.1	50	38.2	0.546
		M	21	60.0	79	63.7		19	67.9	81	61.8	
	A148G PON-2	A	24	68.6	77	62.1	0.479	20	71.4	81	61.8	0.331
		G	11	31.4	47	37.9		8	28.6	50	38.2	
	C311S PON-2	C	5	14.3	30	24.2	0.195	5	17.9	30	22.9	0.551
		S	30	85.7	94	75.8		23	82.1	101	77.1	
T2 (n=143)	Q192R PON-1	Q	23	46.9	41	44.1	0.745	21	42.0	43	46.7	0.587
		R	26	53.1	52	55.9		29	58.0	49	53.3	
	L55M PON-1	L	15	30.0	37	39.8	0.243	18	35.3	34	37.0	0.843
		M	35	70.0	56	60.2		33	64.7	58	63.0	
	A148G PON-2	A	29	58.0	51	54.8	0.716	29	56.9	51	55.4	0.869
		G	21	42.0	42	45.2		22	43.1	41	44.6	
	C311S PON-2	C	11	22.0	20	21.5	0.945	10	19.6	21	22.8	0.653
		S	39	78.0	73	78.5		41	80.4	71	77.2	

Results are expressed as n (%); Comparison between groups using Chi-square test (χ^2) or Fisher's exact test. Abbreviation: PLWH = People Living with HIV-1; n= number of individuals; % = percentage.

Table S5. Association between biochemical variables with viral load, CD4-TL and CD8-TL in the PLWH group.

Groups	*Independent variable*	*Dependent variable*		
		**Viral load (log)**	**CD4-LT**	**CD8-LT**
**T0 (n=33)**	Age (years)	β=-0.020; R^2= 0.030; p= 0.377	β= -0.007; R^2=0.013; p=0.525	β= -0.004; R^2= 0.007; p= 0.646
	Paraoxonase activity (U/L)	β= -0.002; R^2= 0.044; p= 0.244	β= 0.000 ; R^2= 0.000 ; p=0.906	β=-0.001; R^2= 0.045; p=0.235
	CD4-TL (mil/mm³)	β=-0.323 ; R^2= 0.058 ; p= 0.177		β= 0.349; R^2= 0.175; p= 0.016
	CD8-TL (mil/mm³)	β= -0.037; R^2= 0.004 ; p= 0.740	β=0.499 ; R^2= 0.176 ; p= 0.016	
	Viral load (Log)		β= -0.180 ; R^2= 0.058 ; p= 0.177	β= -0.037; R^2=0.004 ; p= 0.740
	Cholesterol (mg/dL)	β=-0.005 ; R^2= 0.049 ; p= 0.335	β= -0.002; R^2= 0.026 ; p= 0.487	β=0.000 ; R^2=0.001 ; p=0.921
	HDL-C (mg/dL)	β= -0.005 ; R^2= 0.012 ; p= 0.635	β=-0.003 ; R^2= 0.000; p=0.990	β= -0.014; R^2= 0.317; p= 0.008
	LDL-C (mg/dL)	β=-0.003 ; R^2= 0.028 ; p= 0.470	β=-0.001 ; R^2=0.005 ; p=0.760	β=0.001 ; R^2= 0.006 ; p= 0.744
	VLDL-C (mg/dL)	β=-0.001 ; R^2=0.000 ; p= 0.941	β=-0.007 ; R^2=0.048 ; p= 0.339	β=0.011 ; R^2= 0.141 ; p= 0.093
	TG (mg/dL)	β=0.000 ; R^2=0.000 ; p= 0.938	β= -0.001; R^2=0.046 ; p=0.348	β=0.002 ; R^2= 0.137 ; p= 0.098
**T1 (n=28)**	Age (years)	β=-0.020; R^2= 0.030 ; p= 0.377	β= 0.011 ; R^2= 0.027 ; p= 0.404	β= -0.006 ; R^2= 0.019 ; p= 0.480
	Paraoxonase activity (U/L)	β=0.001 ; R^2= 0.004 ; p= 0.759	β=0.000 ; R^2= 0.003 ; p= 0.766	β= 0.001 ; R^2= 0.079 ; p= 0.147
	CD4-TL (mil/mm³)	β= -0.702 ; R^2= 0.181 ; p= 0.024		β= 0.313; R^2= 0.280 ; p= 0.004
	CD8-TL (mil/mm³)	β= -0.023; R^2= 0.000 ; p= 0.967	β= 0.494 ; R^2= 0.244 ; p= 0.008	
	Viral load (Log)		β= -0.258; R^2= 0.181 ; p= 0.024	β=-0.003 ; R^2= 0.000 ; p= 0.967
	Cholesterol (mg/dL)	β= 0.000 ; R^2= 0.000 ; p= 0.964	β=0.002 ; R^2= 0.015 ; p= 0.600	β=-0.001 ; R^2= 0.009 ; p= 0.686
	HDL-C (mg/dL)	β= -0.019; R^2= 0.062 ; p= 0.277	β=-0.003 ; R^2= 0.006; p= 0.749	β= -0.002 ; R^2= 0.006 ; p= 0.749
	LDL-C (mg/dL)	β= 0.004 ; R^2= 0.012 ; p= 0.633	β= 0.003; R^2= 0.029 ; p= 0.464	β= -0.002; R^2= 0.000; p= 0.992
	VLDL-C (mg/dL)	β= -0.002 ; R^2= 0.001 ; p= 0.894	β= 0.001; R^2= 0.001 ; p= 0.888	β= -0.003; R^2= 0.023 ; p= 0.509
	TG (mg/dL)	β= 0.000 ; R^2= 0.003 ; p= 0.808	β= -0.008 ; R^2= 0.000 ; p= 0.974	β=0.006 ; R^2= 0.000 ; p= 0.979
**T2 (n=53)**	Age (years)	β=-0.031 ; R^2= 0.065 ; p= 0.072	β= 0.034; R^2= 0.049 ; p= 0.120	β= 0.023; R^2= 0.059 ; p= 0.089
	Paraoxonase activity (U/L)	β= 0.001 ; R^2= 0.029 ; p= 0.231	β= 0.001 ; R^2= 0.005 ; p= 0.624	β= 0.000 ; R^2= 0.001 ; p= 0.873
	CD4-TL (mil/mm³)	β= -0.209 ; R^2= 0.069 ; p= 0.062		β= 0.334 ; R^2= 0.314 ; p<0.001
	CD8-TL (mil/mm³)	β= -0.194 ; R^2= 0.021 ; p= 0.312	β= 0.941 ; R^2= 0.314 ; p<0.001	
	Viral load (Log)		β= -0.332 ; R^2= 0.069 ; p= 0.062	β= -0.110 ; R^2= 0.021 ; p= 0.312
	Cholesterol (mg/dL)	β=-0.005 ; R^2= 0.062 ; p= 0.116	β=0.008 ; R^2= 0.115 ; p= 0.030	β= 0.002 ; R^2= 0.020 ; p= 0.381
	HDL-C (mg/dL)	β=-0.008 ; R^2=0.018 ; p= 0.406	β= 0.016; R^2= 0.044 ; p= 0.187	β= -0.003; R^2= 0.004 ; p= 0.692
	LDL-C (mg/dL)	β= -0.007 ; R^2= 0.072 ; p= 0.090	β=0.009 ; R^2= 0.093 ; p= 0.053	β= 0.002; R^2= 0.011 ; p= 0.529
	VLDL-C (mg/dL)	β= 0.002 ; R^2= 0.001 ; p=0.845	β= 0.006 ; R^2= 0.006 ; p= 0.630	β= 0.012 ; R^2= 0.061 ; p= 0.125
	TG (mg/dL)	β= 0.000 ; R^2= 0.004 ; p= 0.687	β= 0.001; R^2= 0.007 ; p= 0.591	β= 0.001 ; R^2= 0.056 ; p= 0.142

Univariate linear regression parameters: β = represents the regression standardized coefficient, that is, how much the dependent variable changes for each unit of change in the independent variable. R^2 = indicates the proportion of variance in the dependent variable that is explained by the model. P-value = tests the statistical significance of the beta coefficients. that is. whether the relationship between the variables is significant. Abbreviations: n= number of individuals; HDL-C: high density lipoprotein-cholesterol; LDL-C: low density lipoprotein-cholesterol; TG: triglycerides; VLDL-C: very low-density lipoprotein cholesterol.
